# Formal and Informal Social Care in People With Rheumatic and Musculoskeletal Diseases: A Cross‐Sectional Multicenter Survey

**DOI:** 10.1002/acr.80031

**Published:** 2026-05-10

**Authors:** Mehreen Somro, Karen Durrant, William G. Dixon, John McBeth, Alex MacGregor, Max Yates, Jenny H. Humphreys

**Affiliations:** ^1^ Centre for Epidemiology Versus Arthritis, University of Manchester Manchester United Kingdom; ^2^ Centre for Epidemiology Versus Arthritis, University of East Anglia Norwich United Kingdom; ^3^ Northern Care Alliance Salford United Kingdom; ^4^ Manchester NIHR Biomedical Research Centre Manchester United Kingdom; ^5^ Norfolk and Norwich University Hospital Norwich United Kingdom

## Abstract

**Objective:**

Rheumatic and musculoskeletal diseases (RMDs) are leading causes of physical disability, necessitating support with activities of daily living. This study describes social care received by patients with RMDs in two disparate regions of England: Salford (urban) and Norfolk (rural).

**Methods:**

A cross‐sectional survey identified how many patients received care and who provided it as well as care type (formal/informal), frequency, and duration. Participants included (1) patients attending rheumatology outpatient facilities at Salford Royal Hospital and (2) participants with inflammatory polyarthritis enrolled in the Norfolk Arthritis Register (NOAR). Descriptive statistics compared the care received, and a logistic regression model investigated factors associated with receiving help.

**Results:**

The Salford cohort was younger and had a wider range of RMDs compared with NOAR. A significant number of participants in the Salford (85%) and NOAR (38%) groups required help with daily activities. Of those needing help, 61% and 73% received care in Salford and NOAR, respectively, predominantly informally from family and friends. Local authorities delivered social care to just 0.5% of Salford and 1.4% of NOAR participants. Daily care was provided to 43% of Salford and 53% of NOAR participants. Weekly care duration varied: 21% of Salford and 31% of NOAR responders received ≤4 hours, and 19% of Salford and 10% of NOAR responders received >20 hours. Having multiple conditions increased the likelihood of receiving care: Salford odds ratio 1.93 (95% confidence interval [CI] 1.18–3.18) and NOAR odds ratio 3.30 (95% CI 1.45–7.60).

**Conclusion:**

The survey highlights a lack of formal care for patients with RMDs, who mostly rely on undocumented informal care. Patients with multiple conditions often require daily living assistance; rheumatology services should consider incorporating social care reviews as part of routine practice.

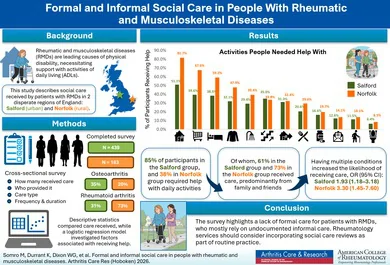

## INTRODUCTION

Rheumatic and musculoskeletal diseases (RMDs) encompass a diverse range of more than 200 conditions that affect the joints, bones, muscles, and tendons and includes both inflammatory and noninflammatory diseases, often resulting in pain, stiffness, and decreased mobility.^1^ The World Health Organization Global Burden of Disease study has highlighted RMDs as a leading cause of disability worldwide, ranking them as the second most prevalent contributor to total years lived with disability.^2^ Within RMDs, rheumatoid arthritis (RA) is a debilitating, multisystem, chronic inflammatory condition and is the most common inflammatory diagnosis within RMDs.^3^ RA has been shown to have a considerable impact on the physical, social, and mental health of patients.^1^ In a recent meta‐analysis, up to 50% of people diagnosed with RA had to stop working because of the disease, leading to a significant decrease in the quality of life, and most are of working age at the time of diagnosis.^4^ Although there has been extensive research into medication to treat active disease, reduce disability, and improve the quality of life for people with RA, despite these advances, many patients still experience significant impairment, known as the disability gap.^5^

**SIGNIFICANCE & INNOVATIONS**

- This study is the first to examine the association between rheumatic and musculoskeletal diseases and social care. Three in five patients received regular care, mostly unpaid from family or friends, revealing a substantial hidden economic burden on patients, caregivers, and society.
- Further, we found that one in three people with these conditions require help with basic activities of daily living such as washing, dressing, and household chores. For 7 of 10 of those receiving this social care, it was on at least a daily basis and often for several hours.
- Importantly, our findings were broadly consistent across two differing regions of the United Kingdom. Norfolk is largely classified by the Office for National Statistics as “majority rural”; by contrast Salford is an urban population. This suggests that these patterns of care do not change based on rurality.

RA and other RMDs lead to substantial economic burdens both directly and indirectly with contributions from both formal and informal care.^6^ Formal caregiving refers to professional health services provided by individuals or agencies that are paid for their care, which can include medical practitioners, in‐home nursing and personal care, and institutional care facilities. In the United Kingdom, health care is provided free at the point of access, through the NHS. In contrast, formal social care is only provided through local authorities on a means‐tested basis; it may also be accessed through self‐funding, charities, and other third‐sector organizations. Informal caregiving, on the other hand, involves unpaid support by family members, friends, or volunteers who provide assistance with daily activities and medical needs. These two types of care contribute to the direct and indirect costs associated with RA and other RMDs. Direct costs encompass medical treatments such as hospital stays, clinic visits, medications, laboratory tests, and imaging procedures, as well as costs for physical therapy and adaptive equipment, which might include alterations to living spaces. These direct expenses are generally well documented.^7^ Indirect costs, on the other hand, stem from decreased work productivity, job‐related disability, and, notably, the necessity for nonprofessional or informal caregiving services. These costs are often more difficult to capture and are typically not well documented in health records.^8^ Support provided by family and friends to those with conditions such as RA and other RMDs is not frequently recognized or recorded as caregiving, making it challenging to assess its economic value. This informal care, although often essential, is difficult to quantify monetarily because of its private and unstructured nature.

Further, caregiving can extend past activities of daily living (ADLs) to include help with mobility, household chores, medication management, and transport. The indirect costs relating to supporting individuals, which can be defined as social care support, have largely been unexplored in people with RMDs and are crucial in determining the true disease burden they pose to individuals. This is in part because this information is not captured routinely and needs to be asked about directly. This study aimed to characterize and quantify both formal (provided by local authorities and other paid care providers) and informal (provided by friends, family, and others) social care in people with RMDs in two distinct regions of the United Kingdom, one rural and one urban.

## PATIENTS AND METHODS

### Population

Two different groups of patients with RMDs were sent an online cross‐sectional survey to identify and characterize formal and informal social care use (details below). The first were unselected adult patients attending Salford Royal Hospital Rheumatology outpatient clinics as part of a wider study nested within the “Data Jigsaw” program of research (https://thedatajigsaw.co.uk/). The survey was distributed via QR codes and text messages, and informed electronic consent was gained. Text message invites were sent to patients who had attended these clinics between October 2022 and September 2023 via the DrDoctor messaging service. Patients were also recruited directly from rheumatology clinics between November 2022 and March 2023. Rheumatology patients at Salford Royal Hospital were eligible to participate if they were adults with a musculoskeletal condition under the hospital's care, had internet access, and could understand English with or without help from a caregiver. Eligibility included adults older than 18 years with a musculoskeletal diagnosis who had seen a rheumatologist at Salford Royal and were under active follow‐up with the rheumatology department.

The second group were current participants of the Norfolk Arthritis Register (NOAR). NOAR has been described in detail elsewhere^9^; briefly, since 1990, NOAR has recruited adults older than age 16 years from Norfolk with recent swelling in two or more joints for at least 4 weeks. Participants who were actively enrolled in NOAR (n = 1,085), had consented to contact, and had provided an email address were sent an email invitation to take part in the social care survey between March 27 and 31, 2023. Reminders were subsequently sent to those who had yet to complete the survey between April 17 and May 12, 2023.

### Survey development and refinement

The survey was originally adapted from an existing social care interview survey developed as part of a project funded by the Nuffield Foundation and the Department of Health^10^ and has been used by our team previously.^11^ It was further refined with input from a patient advisory group to develop a web‐based survey to enhance patient usability and reduce burden using Qualtrics software. The patient advisory group was made up of four patient partners living with RMDs based in Salford who took part in a Patient and Public Involvement workshop in January 2022 that explored this and other aspects of the Data Jigsaw program. They also provided feedback specifically on the survey in a postworkshop questionnaire. The workshop and feedback did not recommend major changes to the survey.

The survey collected data on participant demographics, whether help was required with ADLs (for example washing, dressing, or grocery shopping), which activities these were, who provided it, and the frequency and duration of the help provided. This captured both formal and informal help. Details of demographics, comorbid conditions, and partial postcodes were recorded to allow calculation of the Townsend Deprivation Index. Quintiles were created by setting 0 as the threshold for the Townsend Deprivation level to allow comparison with the national average Townsend Deprivation score.^12^ Additional questions were asked in the NOAR survey pertaining to work (see Supplementary Exhibit 1). The Salford study received ethics approval from the UK Health Research Authority (REC ref 22/WA/0056, IRAS number 306827). NOAR received ethics approval from East of England – Cambridgeshire and Hertfordshire Research Ethics Committee (REC reference 15/EE/0076; IRAS 166048 substantial amendment No. 6, amendment date November 8, 2022).

### Analysis

Data from the Qualtrics survey data were exported to R (version 4.2.1) for analysis and visualization. Detailed descriptive and summary statistics were performed. Data from each cohort were analyzed in parallel because of the differences in recruitment approaches and underlying population differences. To examine representativeness, the demographics of each cohort were compared with its background population (rheumatology outpatients for Salford and the NOAR 2020 population for NOAR).

The type, frequency, and duration of care were quantified in both cohorts, and descriptive comparisons were made between the two cohorts. Logistic regression analysis was conducted to identify factors associated with requiring help. Variables included in the model were age, sex, multiple long‐term condition count (defined as a person who has no underlying health conditions or one, two, or more than three comorbidities), and the Townsend Index as a continuous variable. The data used for the survey are not publicly available without the completion of specific training requirements and information governance checks mandated by the data owners.

## RESULTS

### Demographics

A total of 805 people interacted with the Salford survey. Of these, 502 consented to take part, and 439 completed the survey. The majority, 326 (74%), were female, with a median age of 58 years, and an age range spanning from 21 to 94 years (Table 1). Osteoarthritis was self‐reported by 154 participants (35%) and RA by 136 (31%) (see Table 1). Two hundred eighty‐two (64%) participants reported multiple long‐term health conditions (MLTCs).

**Table 1:** Participant demographics*

	Salford			NOAR		
	Social care			Social care		
	No (n = 65)	Yes (n = 374)	Overall (N = 439)	No (n = 112)	Yes (n = 71)	Overall (N = 183)
Age, y						
Median [min–max]	59.0 [21.0–87.0]	57.5 [21.0–94.0]	58.0 [21.0–94.0]	63.0 [27.0–84.0]	63.0 [27.0–84.0]	63.0 [27.0–84.0]
Sex, n (%)						
Female	50 (76.9)	276 (73.8)	326 (74.3)	72 (64.3)	55 (77.5)	128 (69.6)
Male	13 (20.0)	96 (25.7)	109 (24.8)	40 (35.7)	15 (21.1)	55 (29.9)
Missing	2 (3.1)	2 (0.5)	4 (0.9)	0 (0)	1 (1.4)	1 (0.5)
Diagnosis reported, n (%)^a^						
Rheumatoid arthritis	21 (32.3)	115 (30.7)	136 (31.0)	82 (73.2)	53 (74.6)	135 (73.4)
Osteoarthritis	27 (41.5)	127 (34.0)	154 (35.1)	18 (16.1)	20 (28.2)	38 (20.7)
Psoriatic arthritis	7 (10.8)	48 (12.8)	55 (12.5)	16 (14.3)	5 (7.0)	21 (11.4)
Axial spondyloarthropathy	6 (9.2)	28 (7.5)	34 (7.7)	0 (0)	2 (2.8)	2 (1.1)
Other inflammatory condition	5 (7.7)	32 (8.6)	37 (8.4)	8 (7.1)	7 (9.9)	15 (8.2)
Sjögren disease	5 (7.7)	32 (8.6)	37 (8.4)	40(35.7)	48(67.6)	88(48.1)
Systemic sclerosis	7 (10.8)	38 (10.2)	45 (10.3)	–	–	–
Osteoporosis	10 (15.4)	42 (11.2)	52 (11.8)	9 (8.0)	6 (8.5)	15 (8.2)
Fibromyalgia	19 (29.2)	99 (26.5)	118 (26.9)	15 (13.4)	14 (19.7)	29 (15.8)
Other	15 (23.1)	96 (25.7)	111 (25.3)	54 (48.2)	28 (39.4)	82 (44.8)
Multiple long‐term conditions, n (%)^b^	11 (16)	271 (72)	282 (64)	17 (15.2)	8 (11.3)	25 (13.7)
Townsend Deprivation Index category						
1 (<3.80), n (%)	7 (10.8)	70 (18.7)	77 (17.5)	17 (15.2)	14 (19.7)	31 (16.9)
2 (−3.79 to −2.92), n (%) and median [min–max]	4 (6.2)	18 (4.8)	22 (5.0)	63.0 [27.0–84.0]	63.0 [27.0–84.0]	63.0 [27.0–84.0]
3 (−2.91 to −2.09), n (%)	11 (16.9)	45 (12.0)	56 (12.8)	72 (64.3)	55 (77.5)	128 (69.6)
4 (−2.08 to −0.55), n (%)	20 (30.8)	88 (23.5)	108 (24.6)	40 (35.7)	15 (21.1)	55 (29.9)
5 (>−0.54), n (%)	23 (35.4)	152 (40.6)	175 (39.9)	0 (0)	1 (1.4)	1 (0.5)

* Max, maximum; min, minimum; NOAR, Norfolk Arthritis Register.

^a^ Some participants reported more than one diagnosis for their pain.

^b^ Includes the musculoskeletal disorders above and the following chronic diseases listed in the questionnaire: angina, heart attack, heart disease, anxiety, gastrointestinal disease, stroke, chronic obstructive pulmonary disease, diabetes, cancer, multiple sclerosis, depression, and other.

In NOAR, a total of 1,085 personalized invitation emails were sent between March 27 and March 31, 2023, with 210 individuals taking part. Of these, 183 consented and completed the survey. The majority, 128 (69%), were female, with a median age of 63 years and an age range spanning from 27 to 84 years (Table 1). Because of the population of NOAR, a high proportion of NOAR respondents had a diagnosis of RA (n = 135, 73%). Osteoarthritis was reported by 38 of the participants (20%) (see Table 1). Eighty‐eight participants (48%) reported MLTCs. Thirty‐nine percent of the respondents were in paid employment, 51% were retired, 3% were looking for work, and 7% were unable to work.

The demographics of the Salford survey respondents were similar to those of the hospital population, and the NOAR respondents were similar to the NOAR cohort. This comparison included age, sex, Index of Multiple Deprivation, and ethnicity. The average age for both populations was similar, with a higher proportion of women, and most of the population was of a White British background. Reporting of other ethnicities was not possible because of low counts and the resultant risk of participant re‐identification.

### ADLs

**Figure 1:**
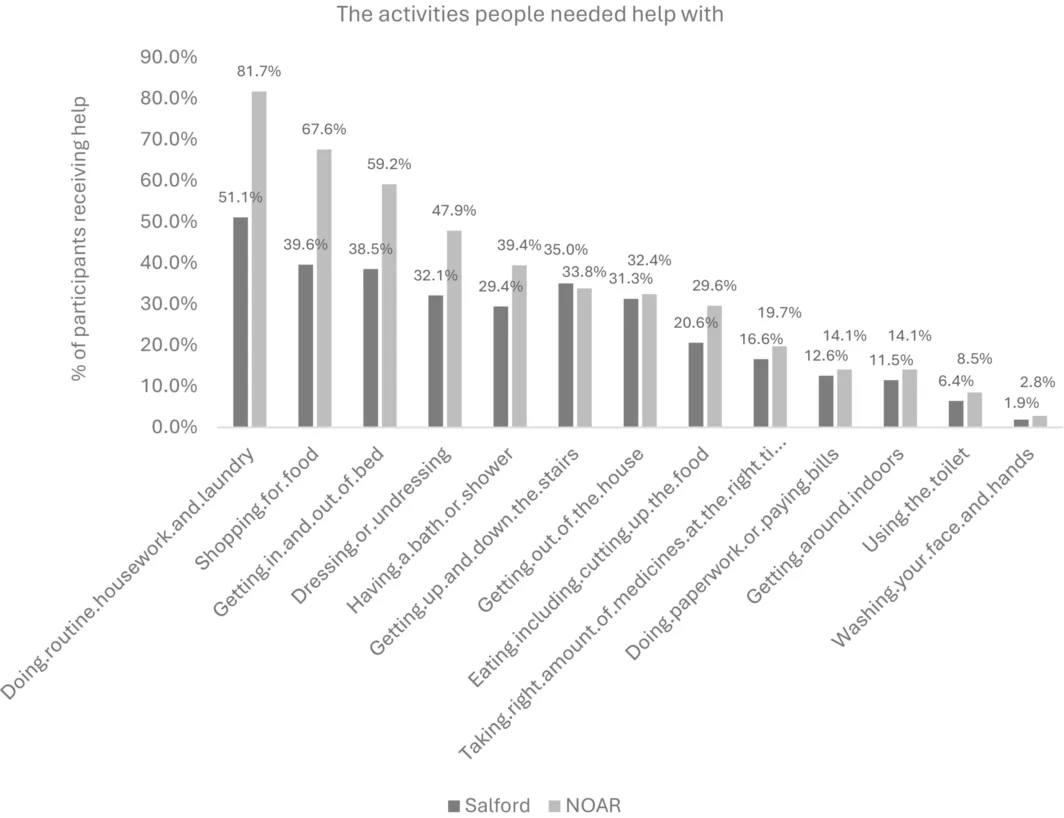
Activities of daily living requiring social care. NOAR, Norfolk Arthritis Register.

People who reported living with an RMD often required assistance with ADLs, with 85% (374 of 439) of the Salford cohort and 38% (71 of 183) of the NOAR cohort stating they required help. These ADLs included carrying out household chores, grocery shopping, and managing to get in and out of bed. Specifically, nearly half of the Salford individuals and more than 80% of the NOAR participants who reported receiving help did so for routine household tasks, whereas approximately two‐fifths of the Salford individuals and two‐thirds of the NOAR participants reporting receiving help did so for grocery shopping. The next most common reported activity with those receiving help was getting in and out of bed. Moreover, approximately one‐third of the Salford participants and almost half the NOAR participants reporting receiving assistance did so for dressing and undressing, approximately one‐third of both cohorts for leaving their homes, and roughly one‐sixth for help with medication (see Figure 1).

### Providers of social care

**Figure 2:**
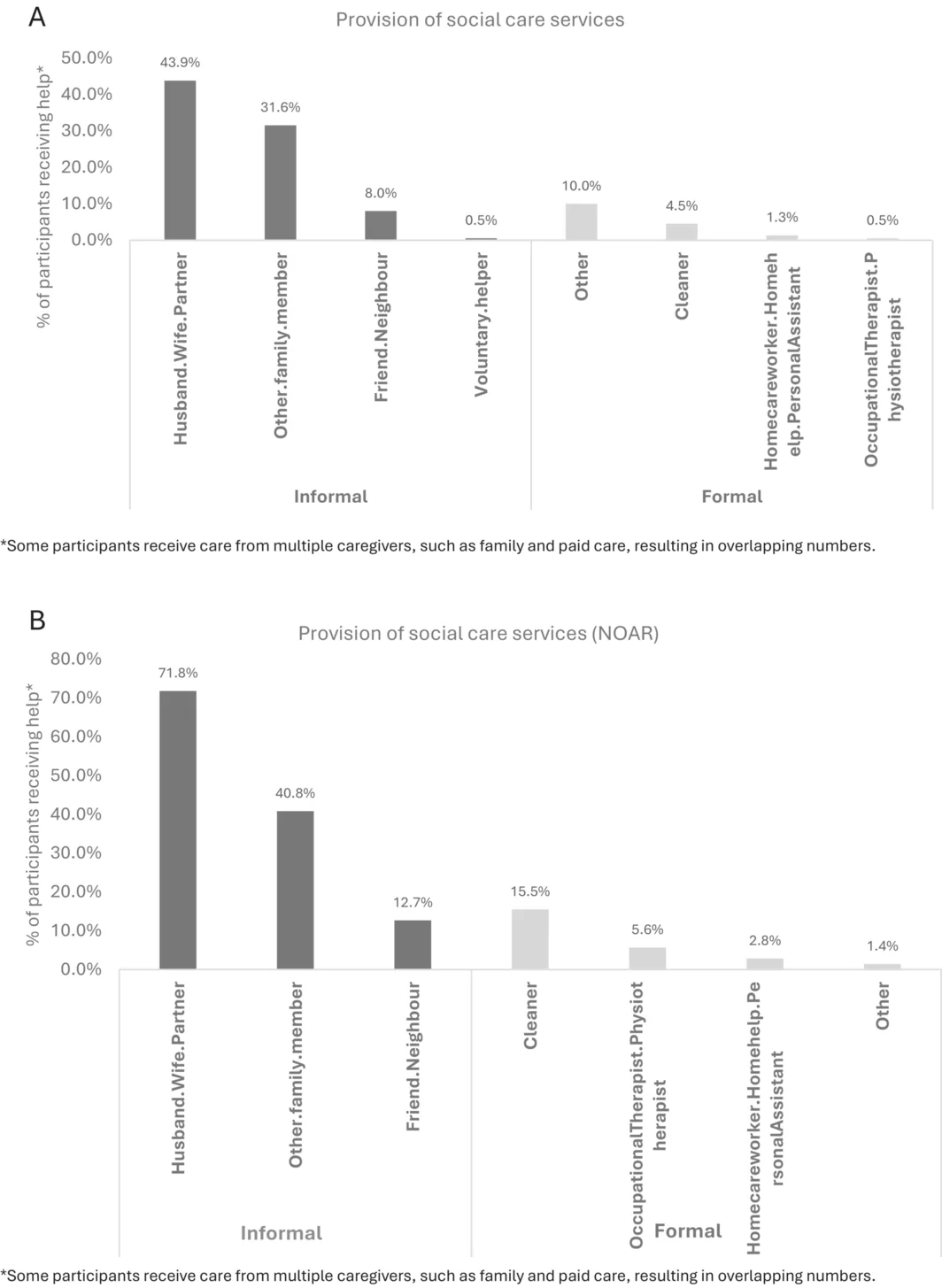
(A) Provision of social care services (Salford). (B) Provision of social care services (NOAR). *Some participants receive care from multiple caregivers, such as family and paid care, resulting in overlapping numbers. NOAR, Norfolk Arthritis Register.

For both groups, most participants who received assistance with ADLs received help informally from family and friends (Figure 2A and B): 61% (226 of 374) in Salford and 73% (52 of 71) in NOAR. Approximately 16% (63 of 374) of Salford participants and 23% (17 of 71) of NOAR participants arranged and paid for assistance themselves without involvement from local authorities or social care organizations. Notably, only a small percentage in both surveys (0.5% or 2 of 374 for Salford and 1.4% or 1 of 71 for NOAR) had their services paid for by the local authority. There were also a few participants (2.4% [9 of 373] for Salford and 8.4% [6 of 71] for NOAR) who received support from homecare assistants, occupational therapists, and voluntary helpers (Figure 2A and B). Overall, 10% of the Salford cohort reported “other,” and the other included a variety of examples such as “gardener,” “hospital staff,” and “supermarket delivery.” A small number of participants received help from more than one group, for example, family and friends as well as paid services: in Salford, this was two individuals, and for NOAR this was 18 people. A total of 16% (63 of 374) also received help with leisure activities, such as hobbies, socializing, or meeting up with friends in the last month.

### Duration and frequency

**Figure 3:**
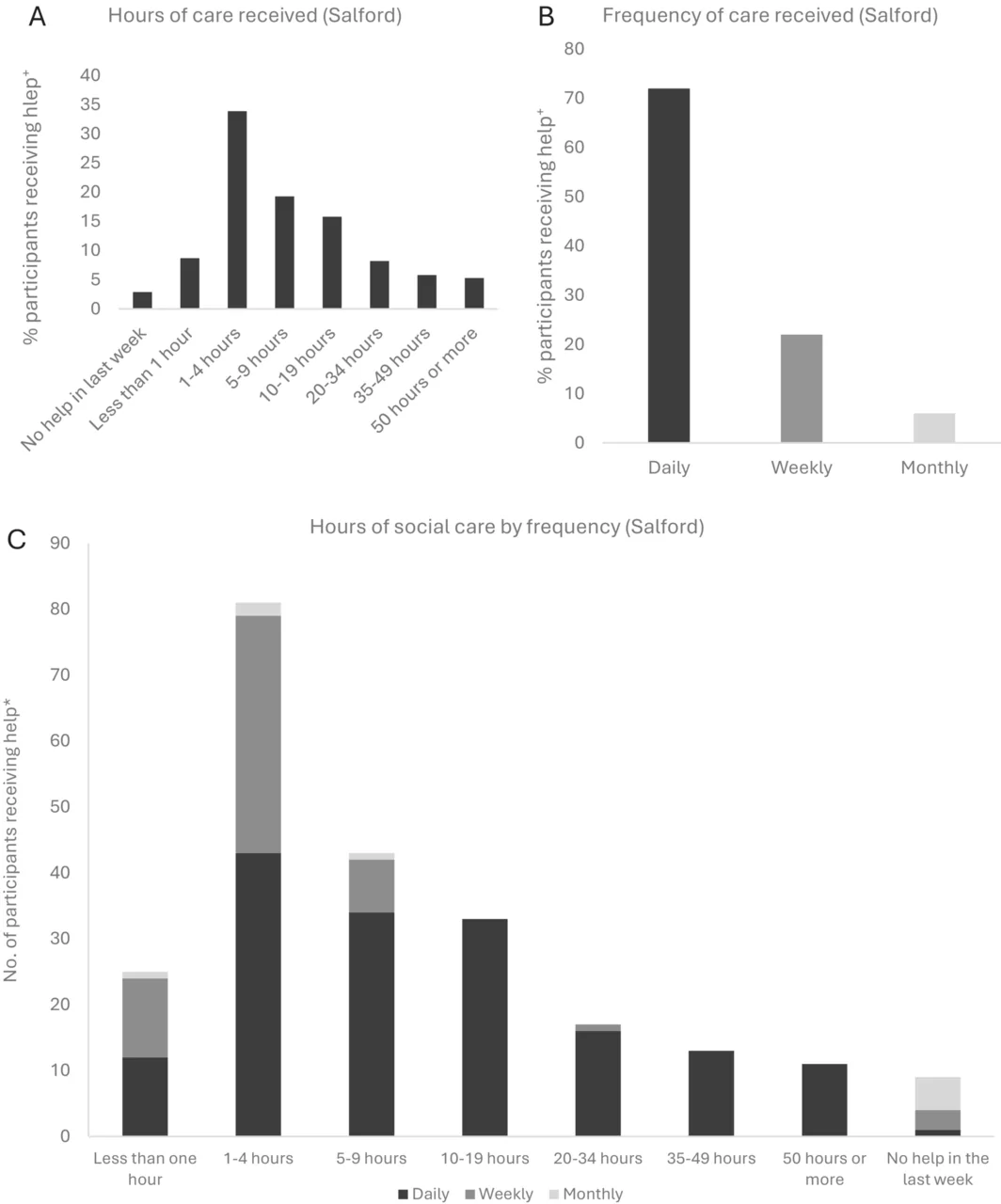
(A) Hours of social care received per week H (Salford). (B) Frequency of social care services received (Salford). (C) Hours of social care by frequency (Salford). +Presented as the percentage of nonmissing data. *Some participants receive care from multiple caregivers, such as family and paid care, resulting in overlapping numbers.

**Figure 4:**
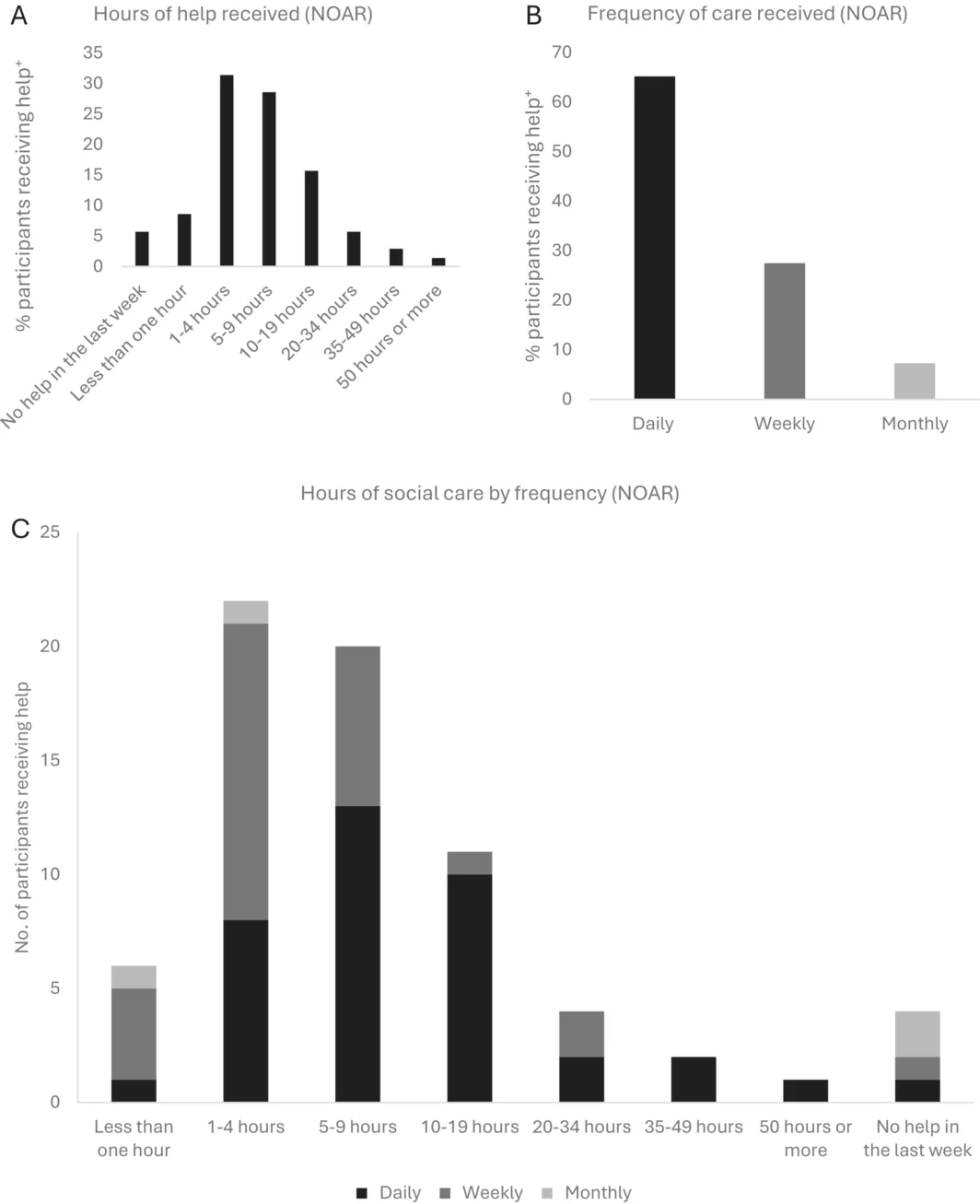
(A) Hours of social care received per week (NOAR). (B) Frequency of social care services received (NOAR). (C) Hours of social care by frequency (NOAR). +Presented as the percentage of nonmissing data. NOAR, Norfolk Arthritis Register.

The amount of time that assistance was received per week varied among the participants, with the largest group receiving care for 4 hours or less per week (46% [80 of 171] of respondents in Salford and 30% [22 of 71] in NOAR cohorts). However, a notable minority (19% [n = 74] in Salford and 25% [n = 18] in NOAR) required more than 10 hours of care per week (Figures 3A and 4A). Approximately 7 in 10 people (72% [n = 123] in Salford and 68% [n = 45] in NOAR) received assistance on a daily basis (Figure 3B and C; Figure 4B and C). Participants who required daily care also tended to require more weekly hours of support (Figures 3C and 4C) compared with those who received care less frequently. There was a significant number of participants in the Salford cohort who did not answer these two questions, with 201 nonresponders. Across both cohorts, the frequency and duration of support was similar between those receiving formal and informal care (Supplementary Figure 1A–D). In those receiving very infrequent or occasional help (once per month or less), it was predominantly provided formally.

### Factors associated with receiving social care

In the two multivariable models (Table 2), similar patterns were identified across both cohorts. Unexpectedly, younger people were found to be more likely to receive help with ADLs in Salford, with an odds ratio (OR) of 0.97 for age (95% confidence interval [CI] 0.96–0.99). However, in the NOAR population, age was not associated with receiving help, with an OR of 0.99 (95% CI 0.97–1.02). Participants who had MLTCs (defined as reporting at least one additional long‐term condition to their index RMD) were found to be more likely to receive help across both cohorts, with one additional long‐term condition OR of 1.93 (95% CI 1.18–3.18) and 3.30 (95% CI 1.45–7.60) in Salford and NOAR, respectively.

**Table 2:** Factors associated with social care received in the last month*

	Salford				NOAR			
	aOR	95% CI		*P*	aOR	95% CI		*P*
Age	0.97	0.96	0.99	<0.001	0.99	0.97	1.02	0.57
Sex								
Female	Ref	Ref	Ref		Ref	Ref	Ref	
Male	0.31	0.19	0.51	<0.001	0.42	0.19	0.88	0.035
Townsend Deprivation Index	1.02	0.93	1.13	0.61	1.04	0.82	1.30	0.76
Additional long‐term conditions								
0	Ref	Ref	Ref		Ref	Ref	Ref	
1	1.93	1.18	3.18	<0.001	3.3	1.45	7.60	<0.001
2	3.76	2.08	6.95	<0.001	5.87	2.53	14.2	<0.001
≥3	2.92	1.55	2.89	<0.001	2.14	1.40	9.89	<0.001

* Multivariate logistic regression, adjusting for all variables presented in the table. aOR, adjusted odds ratio; CI, confidence interval; NOAR, Norfolk Arthritis Register; Ref, reference.

Answering “yes” to the question “Have you received help with activities of daily living in the last month?”

## DISCUSSION

In this survey of people with RMDs in two sites across the United Kingdom, three to four of every five people who received help did so informally without financial remuneration from family and friends. We have shown approximately one in three people with these conditions need help with basic ADLs, such as shopping, household chores, and personal care activities. This help is need frequent and for several hours daily. In addition, people with MLTCs were more likely to need help. Despite the differences in the demographics between the two regions investigated, and in particular rural versus urban settings, the results were broadly similar across the cohorts.

To our knowledge, this is the first study to investigate the relationship between RMDs and social care use. It is an important outcome to describe for people with RMDs because RMDs are the most common cause of chronic physical disability. As a group, they are likely to require support with ADLs at some point during their lifetime. Previously, we have investigated these rates in people with chronic pain and also found high levels of informal social care provision.^11^ According to the Office for National Statistics (ONS), there are approximately five million unpaid carers across England and Wales, representing a significant economic burden.^13^ Understanding which diseases drive the need for care is therefore important. RMDs are often present in people with MLTCs and contribute significantly to function disability in this context.^14^, ^15^ We have demonstrated here that this is also an important factor in driving the social care burden. Further work remains to understand the relative social care burden of RMDs compared with other long‐term conditions. We have also provided some insight into which activities these patients need help with that may be important for patient charities and support groups. The care provided is often highly personal, and, therefore, it may be the informal care that is being required, falling heavily on very close friends and family when professional support is not accessible or available.

In the United Kingdom, government‐funded social care comes under the Care Act 2014 and is provided through local authorities (councils).^16^ This law states eligibility for formal social care has been assessed on the basis of an individual's care needs and is not dependent on other informal care that may be provided. Our study demonstrates that rates of council‐funded or paid‐for care are low in people with RMDs, with less than 2% reporting this in our survey. These low levels of formal care provision suggest that people with RMDs either do not meet the eligibility criteria for formal care or do not seek out formal care support. Nevertheless, significant amounts of unpaid informal care are being provided, demonstrating a large “care gap.” Informal carers are filling this gap with implications for society and the economy. For example, older carers themselves may have their own health needs that are left unattended, or for younger carers there may be significant impact on their education and/or employment. These costs should be considered for inclusion in the health economic analysis of the disease burden from RMDs.

These data provide a snapshot of care use in people with RMDs. The strengths of this study include a large survey of two regions of the United Kingdom across a spectrum of different socioeconomic positions and rurality. Salford is classified by the UK ONS within its most urban regional category. In contrast, Norfolk has a mixed profile: approximately 25% of its population lives in the urban center of Norwich, and the rest falls under rural classifications ranging from “intermediate rural” to “majority rural,” with the latter being the most rural category defined by the ONS.^10^ The population of Salford is younger, with a median age of 34 years; in comparison, the North Norfolk local authority has the highest median age in England at 54 years. Both regions have a higher proportion of population of White ethnicity compared with England and Wales as a whole.^17^ Salford has high levels of deprivation and is in the top 5% of the most deprived local authorities in England.^18^ By contrast Norfolk is in the bottom 50% of the least deprived regions.^19^

Importantly, the study measured both formal and informal care, allowing for the quantification of the hidden burden of informal care in this patient group. However, there are some limitations. We remain unsure of the prevalence of social care use at a population level because of response rates and possible selection bias in who responded to the survey. The NOAR survey recruited people from a pre‐existing study of patients with inflammatory arthritis. We have compared the demographics in both the rheumatology outpatient population in Salford and the overall population in NOAR (data not shown), and these appear similar. However, we cannot rule out unmeasured differences between those who responded to the survey and those who did not. We identified an association between younger age and receiving help among Salford respondents; this somewhat surprising finding may also be because of a response or selection bias, as younger people with more significant needs may have been more likely to take part in the survey.

It is possible that people invited but not responding to the study were either more or less likely to require help. This could be because rates of digital literacy are associated with markers of deprivation, and therefore, those not responding may have been in need. Although the participants represented an economically diverse group, the study population had little ethnic diversity, with an underrepresentation of non‐White participants compared with the UK population in general. This is in keeping with the demographics of the two regions recruited from (Salford and Norfolk), which limits the applicability of our findings to other ethnic groups, which may access and provide care differently. Furthermore, a cross‐sectional study is not suited to address whether these arrangements have changed over time. Differential selection bias may be present because of the distinct characteristics of the Salford social care survey respondents compared with the NOAR research cohort. The Salford respondents, having actively participated in a survey focused on social care, might exhibit different response tendencies or perspectives compared with the NOAR cohort accustomed to a broader range of survey topics. This potential disparity in response behaviors could introduce bias, influencing the generalizability of findings from either group to the broader population.

In conclusion, there is a large care gap among this population of patients, which has significant ramifications for future care policy. With increasing rates of RMDs, this care gap will affect larger proportions of the population with consequences for the health and productivity of informal caregivers. By capturing informal care use by patients living with RMDs, this study highlights and quantifies this problem. This is crucial as these data are currently not captured by administrative organizations. Requiring assistance with ADLs was associated with MLTCs and those of younger age. Policymakers and care providers, as well as clinicians providing care to patients with RMDs, should consider these results in their policymaking and practice and be aware of the high unmeasured burden of informal care in this population. It might be possible to consider incorporating an annual assessment of formal and informal care provision for patients within routine rheumatology services, supported by the full multidisciplinary team. This could allow signposting to available support and services as well as providing an opportunity for more detailed data collection to better define need and refine care provision in this population.

## AUTHOR CONTRIBUTIONS

All authors contributed to at least one of the following manuscript preparation roles: conceptualization AND/OR methodology, software, investigation, formal analysis, data curation, visualization, and validation AND drafting or reviewing/editing the final draft. As corresponding author, Dr Humphreys confirms that all authors have provided the final approval of the version to be published and takes responsibility for the affirmations regarding article submission (eg, not under consideration by another journal), the integrity of the data presented, and the statements regarding compliance with institutional review board/Declaration of Helsinki requirements.

## Supporting information


**Disclosure Form**:

**Supplementary figure 1 (a)** Hours of care by formal/informal providers (Salford). **(b)** Frequency of social care provision by formal/informal providers (Salford). **(c)** Hours of care by formal/informal providers (NOAR). **(d)** Frequency of social care provision by formal/informal providers (NOAR).

**Supplementary 1**:
