# Supplementary material for: Formal and Informal Social Care in People With Rheumatic and Musculoskeletal Diseases: A Cross‐Sectional Multicenter Survey
Source: Arthritis Care Res (Hoboken). 2026 May 10;78(10):1522–31. doi: 10.1002/acr.80031 (PMC13613254; doi:10.1002/acr.80031)
Supplement: Supplementary file 2 [file ACR-78-1522-s002.docx]

Hours of care provision (Salford)

% participants receiving help

|  | Informal | Formal |
| --- | --- | --- |
| No help in the last week | 3.6 | 4.6 |
| Less than one hour | 10.7 | 4.6 |
| 1-4 hours | 34.2 | 36.3 |
| 5-9 hours | 19.1 | 13.6 |
| 10-19 hours | 14.2 | 13.6 |
| 20-34 hours | 7.6 | 13.6 |
| 35-49 hours | 5.8 | 9.1 |
| 50 hours or more | 4.9 | 4.6 |

Hours of care provision (NOAR)

% participants receiving help

|  | Informal | Formal |
| --- | --- | --- |
| No help in the last week | 6.3 | 0 |
| Less than one hour | 9.4 | 6.7 |
| 1-4 hours | 31.3 | 46.7 |
| 5-9 hours | 28.1 | 40 |
| 10-19 hours | 14.1 | 6.7 |
| 20-34 hours | 6.3 | 0 |
| 35-49 hours | 3.1 | 0 |
| 50 hours or more | 1.6 | 0 |

Frequency of care provision (Salford)

% participants receiving help

|  | Informal | Formal |
| --- | --- | --- |
| Daily | 72 | 59.1 |
| Weekly | 23.1 | 27.3 |
| Monthly | 4.88 | 13.6 |

Frequency of care provision (NOAR)

% participants receiving help

|  | Informal | Formal |
| --- | --- | --- |
| Daily | 66.7 | 50 |
| Weekly | 27 | 28.6 |
| Monthly | 6.4 | 21.43 |

**Supplementary figure 1: (a)** Hours of care by formal/informal providers (Salford)**.** **(b)** Frequency of social care provision by formal/informal providers (Salford). **(c)** Hours of care by formal/informal providers (NOAR). **(d)** Frequency of social care provision by formal/informal providers (NOAR).
