# Supplementary material for: Formal and Informal Social Care in People With Rheumatic and Musculoskeletal Diseases: A Cross‐Sectional Multicenter Survey
Source: Arthritis Care Res (Hoboken). 2026 May 10;78(10):1522–31. doi: 10.1002/acr.80031 (PMC13613254; doi:10.1002/acr.80031)
Supplement: Supplementary file 3 [file ACR-78-1522-s003.docx]

Questionnaire on social care usage for participants The Norfolk Arthritis Register

This questionnaire is about how people with inflammatory arthritis get help with their day to day lives.

Please confirm you give consent to take part in this questionnaire by ticking this box

Section 1:

1. Age (years)
2. Gender
3. First half of home postcode
4. Please indicate your occupational status (please tick one box):

| Employed full time (35/37.5 or more hours per week) |  |
| --- | --- |
| Employed part time (up to 35/37.5 hours per week) |  |
| Unemployed and currently looking for work |  |
| Unemployed and currently not looking for work |  |
| Student |  |
| Retired |  |
| Homemaker |  |
| Self-employed |  |
| Unable to work |  |

5. Has your doctor given you a diagnosis for your joint pain or swelling?

Yes/No

6. If yes, PLEASE CHOOSE ALL THAT APPLY:

| Rheumatoid arthritis |  |
| --- | --- |
| Psoriatic arthritis |  |
| Ankylosing spondylitis/ spondyloarthropathy |  |
| Inflammatory arthritis (type not specified) |  |
| No medical diagnosis |  |
| Other (please specify): |  |

7. Please indicate if you have/have had any of the following medical conditions: PLEASE CHOOSE ALL THAT APPLY

| Angina |  |
| --- | --- |
| Heart attack |  |
| COPD |  |
| Stroke |  |
| Diabetes |  |
| Cancer |  |
| Parkinson’s |  |
| Multiple Sclerosis |  |
| Depression |  |
| Other (please specify): |  |

Section 2:

The next set of questions are about help you have received in the **last month** with any of the following tasks, even if you don’t usually need help:

- Having a bath or shower
- Getting in and out of bed /washing your face and hands /dressing or undressing/ using the toilet/ eating, including cutting up food/ taking the right amount of medicine at the right times/ getting around indoors/ getting up and down stairs/
- Getting out of the house / Shopping for food / Doing routine housework or laundry/ doing paperwork or paying bills

8. Have you received help from anyone with the tasks listed above in the **last month**? Include help even if it was for a short time or provided by husband/ wife/ another family member.
   - Yes
   - No
9. How many of the tasks listed above did you need help with?
10. Which of the tasks listed above did you need help with?

- Having a bath or shower
- Getting in and out of bed
- Washing your face and hands
- Dressing or undressing
- Using the toilet
- Eating, including cutting up food
- Taking the right amount of medicine at the right times
- Getting around indoors
- Getting up and down stairs
- Getting out of the house
- Shopping for food
- Doing routine housework or laundry
- Doing paperwork or paying bills

11. Do you receive this help with these activities because of your arthritis?
    - Yes for some or all
    - No, none of this help is because of my arthritis (go to Q18)
12. Who helped with these tasks in the last month? PLEASE CHOOSE ALL THAT APPLY
    - Husband/Wife/Partner
    - Other family member
    - Friend/Neighbour
    - Home care worker /Home help/Personal Assistant
    - A member of the reablement / intermediate care staff team
    - Occupational Therapist / Physiotherapist
    - Voluntary helper
    - Warden / Sheltered housing manager
    - Cleaner
    - Other (please specify)
    - None of the above
13. Thinking about the person/people who help, in the last week how many hours **in total** have they helped you in person? Please only think about the hours they were helping you with these kinds of tasks and not about the time they were around in the house or there to help you if you needed it.
    - No help in the last week
    - Less than one hour
    - 1-4 hours
    - 5-9 hours
    - 10-19 hours
    - 20-34 hours
    - 35-49 hours
    - 50 hours or more
14. Still thinking only about help received in person, how often does the person/people who help usually help you? (not help over the phone or by internet)
    - More than once a day
    - Daily
    - Two or more times a week
    - Once a week
    - Once a fortnight/ every two weeks
    - Once a month
    - Less than once a month

Section 3:

Still thinking about the help you received in person, was this help paid for?

- Yes
- No

The following section is about paying for help you receive. If the person /people who help you are family/friends/neighbours and you do not pay them, please go to Q18.

15. How was your help arranged?
    - Arranged without involvement from the local authority, council or social service
    - Local authority, council or social services arranged this help for me
    - Local authority, council or social services told me about the help but I arranged it myself or my family arranged it for me
    - Other
    - Don’t know
16. How much money do you pay for the help given PER WEEK? Please enter amount in pounds and pence (estimate if you are not able to give the exact amount):
    - Amount: £
    - Do not wish to answer
    - Not applicable
17. As far as you are aware, does anyone else or any organisation pay or give money to the person/people who help for the care you receive - for example the council/local authority or a family member? Do not count any benefits such as Carers Allowance or Attendance Allowance.
    - Yes, the council / local authority /social services
    - Yes, a family member (with their own money)
    - Yes, other
    - No, nobody else pays
    - Do not wish to answer

Section 4:

The following section is about the type of social care you receive e.g. services, aids or equipment.

18. In the last month, have you regularly used any of the following services?

If yes, PLEASE CHOOSE ALL THAT APPLY

- Attend a Day Centre or lunch club run by the council or a voluntary body
- Meals on Wheels
- Private frozen meal provider such as Wiltshire farm foods
- Food delivery service by supermarket or milkman
- Family, friend or neighbour brought shopping to my house

19. Have you used or received any equipment or adaptations such as those below to help you with daily living, irrespective of who supplied it? PLEASE CHOOSE ALL THAT APPLY
    - An alarm which you can use to call for help (worn around neck or pull cord in your home)
    - Stairlift
    - Indoor or outdoor rails (e.g. grab rail)
    - Bathroom adaptations (toilet equipment/commode/walk in shower/over bath shower)
    - Hoist
    - Other (please specify)
    - None of the above
20. Do you currently use any of these to help with mobility? Items used indoors and/ or outdoors should be included. PLEASE CHOOSE ALL THAT APPLY
    - A manual or electric wheelchair
    - A mobility scooter
    - Elbow crutches or walking stick
    - Zimmer frame or other walking frame
    - Other (please specify)
    - None of these
21. Do you currently live in any of these types of accommodation?
    - Warden maintained or sheltered accommodation
    - Housing with care, extra care housing or very sheltered accommodation
    - Residential care e.g. nursing home or care home
    - Other type of supported housing (Please specify)
    - None of the above

Please not that once you have submitted the completed questionnaire, your data will be anonymised and it will no longer be possible to withdraw from the study.

Thank you for your participation. If you would like to know more about what social care services your local council provides or you may be eligible for, the following websites may be useful:

NHS:

- <https://www.nhs.uk/conditions/social-care-and-support-guide/help-from-social-services-and-charities/>

GOV.UK:

- <https://www.gov.uk/apply-needs-assessment-social-services>
- <https://www.gov.uk/day-care-centres>
- <https://www.gov.uk/meals-home>

In addition your local NHS service, [Citizens Advice Bureau](https://www.citizensadvice.org.uk/) or a charity might be able to help.

Versus Arthritis have resources and information for carers of people with arthritis here:

<https://www.versusarthritis.org/about-arthritis/living-with-arthritis/carers/>

The following document also takes you through eligibility, assessment and provision of care for people with arthritis:

<https://www.versusarthritis.org/media/11747/know-your-rights-social-care-19.pdf>
